# A Conversation with Elaine Bearer, Neuropathologist

**DOI:** 10.1021/acscentsci.5c00840

**Published:** 2025-05-19

**Authors:** Louisa Dalton

## Abstract

Microplastics in dementia-affected brains resisted identification until she lit them up.

## Graphical abstract

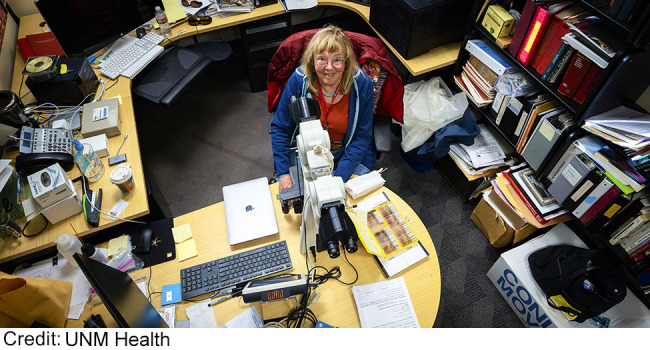

When neuropathologist Elaine Bearer first encountered brown deposits in two human cadaver brains from people who had dementia, she could not identify them. None of her conventional stains or other imaging techniques revealed what the glassy blobs might be.

A few months later, in May 2024, Bearer, a professor at the University of New Mexico, heard that fellow UNM researcher Matthew Campen was using pyrolysis gas chromatography/mass spectrometry to test for microplastics in brain tissue. Suspecting the presence of plastics in her dementia-affected brains, she sent a sample to his group. The results were striking. The dementia-associated tissue had five times as much microplastics as the nondementia-associated samples they had been studying.

But Bearer still did not know whether her brown lumps were plastics. Other pathologists had been seeing similar deposits in other brains, but no one had yet worked out a way to identify them.

Fortunately, innovation in neuroimaging is Bearer’s specialty. She and her colleagues got to work trying myriad stains and instruments to identify the brown spots.

In fall 2024, they developed a microscopy method that finally confirmed that they were looking at microplastics in brain samples. Now she wants to use magnetic resonance imaging (MRI) to track microplastics in living brains and help discover whether plastics could be linked to neurodegenerative diseases.

Louisa Dalton spoke with Bearer about her quest to help pathologists see microplastics in the brain and the further questions their presence raises. This interview was edited for length and clarity.

**Figure:**
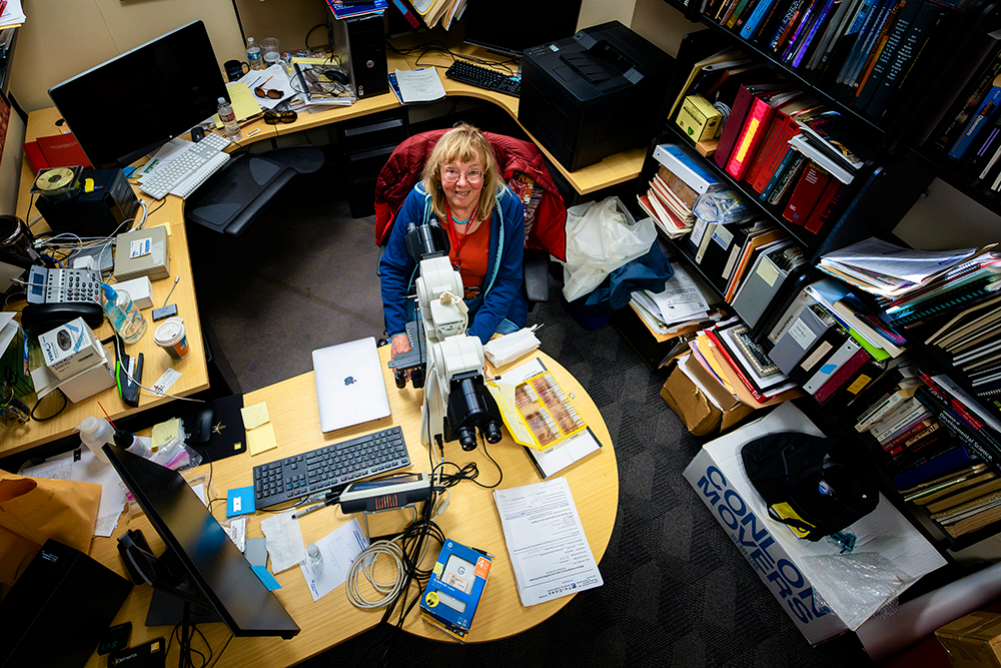
Elaine Bearer sits at her “wonderful” pathology light microscope in her office at the University of New Mexico. Credit: UNM Health.

## Is this the first time pathologists have identified these brown deposits in brains as plastics?

Yes, this is the first time ever! And I’ve been a neuropathologist for what, 30 years? And I do not know why I just noticed them for the first time in these brains. I think it is because when they’re aggregated, they look like brown deposits under a light microscope, but nothing stains them, and all the individual particles are too small to resolve with a light microscope.

## What did you do after realizing your brains from people with dementia had far more plastic deposits than healthy brains?

At first, I was not yet sure that the plastics Matt purified were the brown lumps that did not stain. It made sense they would be, but I did not have any chemical way to prove it. It was just a visual correlation.

So the next thing I did was electron microscopy (EM) on the plastic particles that Matt isolated from the brain tissue so I would know what the particles in the tissue should look like.

And I saw little hooks—very, very tiny, 2 nm dimension little hooks with very sharp points.

**Figure:**
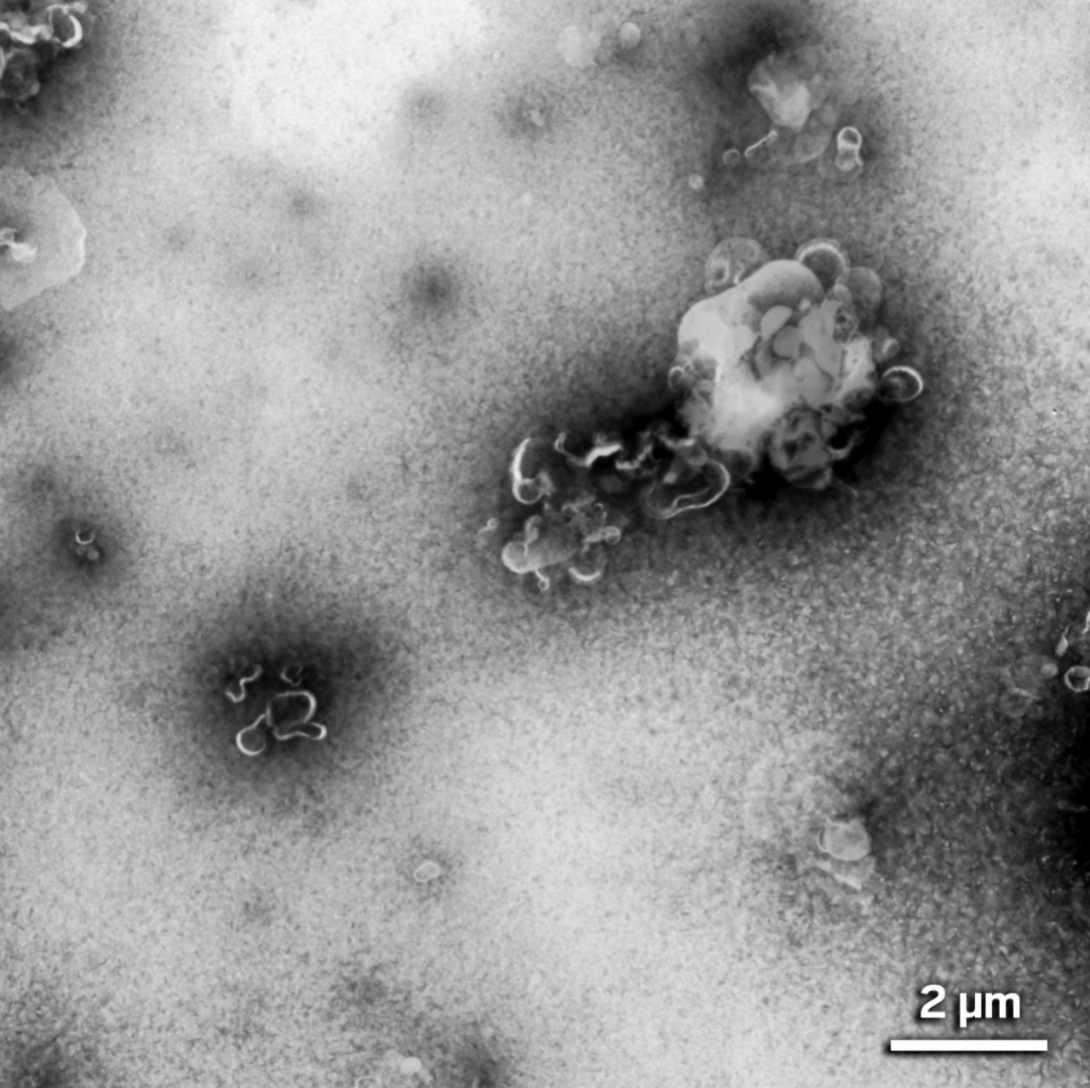
Elaine Bearer found that microplastic clumps isolated from brain samples are made of hook-shaped nanoplastics. Credit: Elaine Bearer.

## Are we talking virus size here? That small?

Oh yes, even smaller. Some of them might be like DNA size, or actin or microtubule size. My friends have told me I should call them micro/nanoparticles because they’re really nanometer size, not micrometer size.

The individual nanoplastics are too tiny to resolve with the optics that we typically use for pathology. So we have not even been seeing most of the plastics in the brain.

## After seeing the isolated nanoparticles, how did you then illuminate and identify them within the tissue?

Yes, our big question last May through August was, “How can we visualize where these plastics are?”

I must have tried 17 different chemical stains. I tried nine different antibody stains. I tried to do EM on brain sections, but you could not see them on sections.

The problem is that when you do EM on sections, you embed your sample in plastic. So you have plastic and plastic together—plastic deposits in your sample touching the plastic you used for the embedding—and you cannot get any contrast.

Finally, I was at [the California Institute of Technology] for a month, and nothing had worked yet. I could not see the plastic nanoparticles in the tissue. I was very frustrated.

I had the isolated plastics on a slide, and I had sections of the brain that they came from. I went down to the laser imaging facility that had a fabulous laser scanning confocal microscope. I’m really good friends with the guy who runs it, and I said, “Is there some optical way that I could maybe see these plastics?”

He tried polarized light microscopy, and we could not see it. But then we tried the confocal. We used 10 different lasers and eight different detectors until we found a way to excite the plastics at one wavelength and have them emit light at a longer wavelength.

We lit it up, and there they were!

**Figure:**
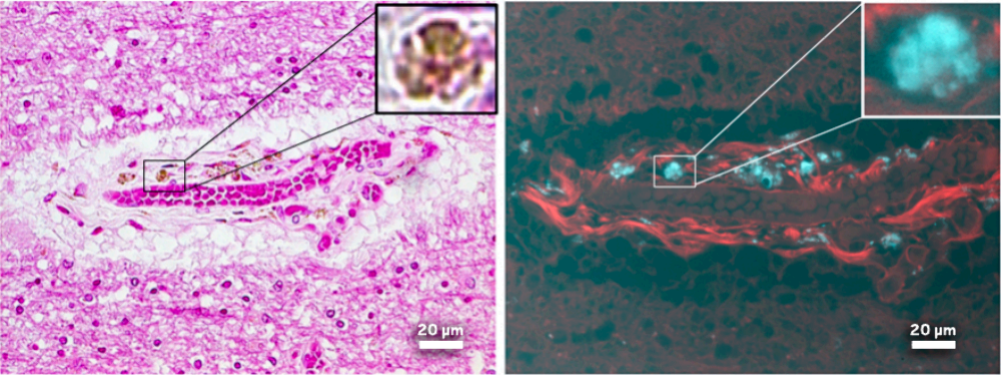
Under a bright-field microscope, brown deposits are visible in this brain sample from a person who had early onset Alzheimer’s disease (left). Using ultraviolet fluorescence microscopy (right), Bearer was able to highlight these deposits (blue) and identify them as microplastics. Credit: Elaine Bearer.

## Is there a link between dementia and the amount of microplastics in the brain?

The abundance seems to be correlated with dementia, and we will now be able to study what they’re doing to the brain. But I do not automatically assume the plastics are bad. They may not be causing disruption of blood vessels.

## What is next?

Next we’re going to see if we can image microplastics in living brains using MRI. And whenever we find them, we’re going to do magnetic resonance spectroscopy, which is a way to detect the molecular composition of the voxels, or 3D pixels, that are giving you the signal.

So maybe, maybe we will get to a point where we can see, in life, what a person’s plastic burden is, and where it is located. If we ever are going to get rid of the plastics or decrease our load or decrease our ingestion, we have to have some measure in a living person. But we have not started this. This is pie in the sky.

## You are director of the New Mexico Brain Bank and lead for the Neuropathology Core at the New Mexico Alzheimer’s Disease Research Center, so I know you have access to a lot of brain samples. You specifically studied these two brains from people who had dementia, but have you now looked at more brains?

Oh yes, 10. And we have not found any brains that do not have any plastics.

All my brains are contemporary, but I also have pathology associates who used to be at Walter Reed [National Military Medical Center], where they have a huge brain collection, some from World War I. Looking at these brains and seeing whether they have plastics or not could be very interesting.

## You also regularly compose and perform music. Are you working on a composition now?

I cannot stop working on compositions, because when my brain is relaxing, I hear music. Right now, I have requests for two pieces. One is an elegy for the Eaton fire. That one I’m already hearing a lot.

I studied composition with Nadia Boulanger when I was a teenager, and she would quote philosophers often. One quote was, “The composer knows when they get it right, because they get a feeling, and that feeling is joy.” And I think in science, it is the same thing. I feel like a musician when I’m doing imaging. We know that we’re seeing an accurate picture of the biology when it looks beautiful and we’ve got it right.

*Louisa Dalton is a freelance contributor to* Chemical & Engineering News, *an independent news publication of the American Chemical Society*.

